# Determination of Capsaicin and Dihydrocapsaicin in *Capsicum* Fruit Samples using High Performance Liquid Chromatography

**DOI:** 10.3390/molecules16108919

**Published:** 2011-10-24

**Authors:** Zeid Abdullah Al Othman, Yacine Badjah Hadj Ahmed, Mohamed Abdelaty Habila, Ayman Abdel Ghafar

**Affiliations:** Department of Chemistry, College of Science, King Saud University, P.O. Box 2455, Riyadh 11451, Saudi Arabia

**Keywords:** *Capsicum*, capsaicin, chilli, pepper, daily capsaicin intake, Scoville heat units

## Abstract

The aim of the present study was to determine the content of capsaicin and dihydrocapsaicin in *Capsicum* samples collected from city markets in Riyadh (Saudi Arabia), calculate their pungency in Scoville heat units (SHU) and evaluate the average daily intake of capsaicin for the population of Riyadh. The investigated samples consisted of hot chillies, red chillies, green chillies, green peppers, red peppers and yellow peppers. Extraction of capsaicinoids was done using ethanol as solvent, while high performance liquid chromatography (HPLC) was used for separation, identification and quantitation of the components. The limit of detection (LOD) of the method was 0.09 and 0.10 µg/g for capsaicin and dihydrocapsaicin, respectively, while the limit of quantification (LOQ) was 0.30 and 0.36 µg/g for capsaicin and dihydrocapsaicin, respectively. Hot chillies showed the highest concentration of capsaicin (4249.0 ± 190.3 µg/g) and the highest pungency level (67984.60 SHU), whereas green peppers had the lowest detected concentration (1.0 ± 0.9 µg/g); green peppers, red peppers and yellow peppers were non pungent. The mean consumption of peppers for Riyadh city population was determined to be 15.5 g/person/day while the daily capsaicin intake was 7.584 mg/person/day.

## 1. Introduction

Capsaicinoids are the compounds responsible for the pungency of pepper fruits and their products. Peppers are the fruits of plants from the genus *Capsicum* and belong to the family *Solanaceae*. There are several domesticated species of chili peppers, among them *Capsicum annuum*, *C. frutescens* and *C. chinense*, which include many common varieties. These various peppers are widely used in many parts of the world for their valued and characteristic sensory properties: color, pungency and aroma. Pungency, a commercially important attribute of peppers, is due to the presence of chemicals from the characteristic capsaicinoids group [1]. The two most abundant capsaicinoids in peppers are capsaicin (8-methyl-*N*-vanillyl-*trans*-6-nonenamide) and dihydrocapsaicin, both constituting about 90%, with capsaicin accounting for ~71% of the total capsaicinoids in most of the pungent varieties [2]. Capsaicin content of peppers is one of the major parameters that determine its commercial quality [3,4,5,6].

Capsaicin is also considered as an active principle which accounts for the pharmaceutical properties of peppers. It has been used as an analgesic against arthritis pain and inflammation [7]. It has also been reported to show anticancer effect [8] and to be active against neurogenic inflammation (burning and stinging of hands, mouth and eyes) [9]. The latter property is the basis for the use of capsaicin in defensive pepper sprays. Capsaicin has also been reported to show protective effects against haigh cholesterol levels and obesity [10]. Capsaicin and other members of the capsaicinoids group produce a large number of physiological and pharmacological effects on the gastrointestinal tract, the cardiovascular and respiratory system as well as the sensory and thermoregulation systems. These effects result principally from the specific action of capsaicinoids on primary afferent neurons of the C-fiber type. This specific influence provides the rationale for their use to treat some peripheral painful states, such as rheumatoid arthritis [11,12,13,14,15,16,17,18]. However, high levels of capsaicin lead to negative health impacts. In a case-control study in Mexico-City which included 220 cases of gastric cancer and 752 controls randomly selected from the general population, chili pepper consumers were at a 5.5-fold greater risk for gastric cancer than non-consumers. Persons who rated themselves as heavy consumers of chili peppers were at an even higher 17-fold greater risk. However, when chili pepper consumption was measured as frequency per day, no significant dose to response relationship was observed [19].

The amount of capsaicin in a given variety can vary depending on the light intensity and temperature at which the plant is grown, the age of the fruit, and the position of the fruit on the plant. The first test developed to measure pungency was the Scoville test, first developed in 1912 by Wilbur Scoville [20]. There are five levels of pungency classified using Scoville heat units (SHU): non-pungent (0–700 SHU), mildly pungent (700–3,000 SHU), moderately pungent (3,000–25,000 SHU), highly pungent (25,000–70,000 SHU) and very highly pungent (>80,000 SHU) [21]. Nowadays, however, the Scoville organoleptic test has been largely replaced by chromatographic methods which are considered to be more reliable and accurate [22]. Capsaicinoids are mainly ingested as naturally occurring pungency-producing components of *Capsicum* spices (chili, cayenne pepper, red pepper). Their concentrations typically range from 0.1 mg/g in chili pepper to 2.5 mg/g in red pepper and 60 mg/g in oleoresin red pepper [23]. Pepper varieties from *Capsicum annuum*, *C. frutescens* and *C. chinense* were found to contain 0.22–20 mg total capsaicinoids/g of dry weight [24]. In another study, cayenne pepper samples had mean capsaicin and dihydrocapsaicin contents of 1.32 and 0.83 mg/g dry weight, respectively [25]. The mean consumption of *Capsicum* spices was reported to be 2.5 g/person/day in India, 5 g/person/day in Thailand [26] and 20 g/person (corresponding to one chili pepper) per day in Mexico [19]. Assuming a content of capsaicinoids in these spices of about 1%, the daily intake of capsaicinoids in these countries has been estimated to 25–200 mg/person/day, which corresponds in the case of a person with 50 kg body weight to 0.5–4 mg/kg bw/day [27]. The maximum daily intake of capsaicin in the U.S. and Europe from mild chilies and paprika was estimated to be roughly 0.025 mg/kg bw/day [28], which is equivalent to 1.5 mg/person/day. According to a recent estimation, the mean and maximum intakes of capsaicin from industrially prepared food products containing the recommended general limit of 5 µg/g, would be 0.77 and 2.64 mg/day, respectively [29].

Kingdom of Saudi Arabia (KSA) imports a lot of foodstuffs from several countries. These food products may be grown in different areas and exposed to different environmental conditions: soil composition, irrigation water and light density; so the objective of this work was to estimate the levels of capsaicin and dihydrocapsaicin that may be present in some pepper samples available in local markets in Riyadh city. These concentrations should allow us to calculate the Scoville heat units (SHU), determine the pungency level of each type of pepper analyzed, evaluate the Riyadh population daily intake of capsaicin, then compare the results with other studies carried out around the world to know whether these levels exceed the accepted international range.

## 2. Results and Discussion

**Figure 1:**
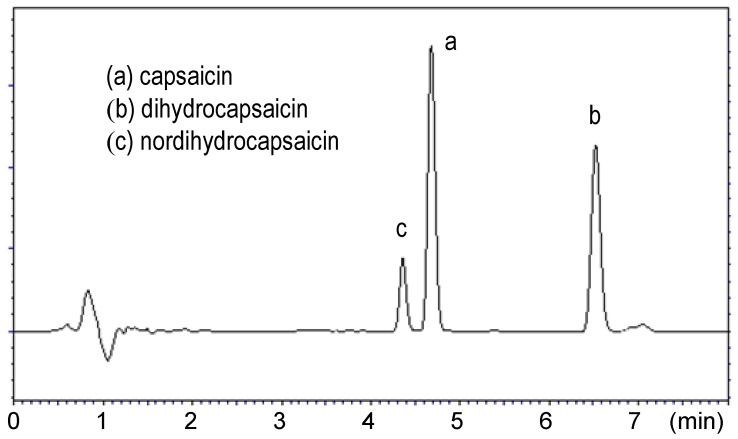
Chromatogram of the standard solution corresponding to 100 µg/g of capsaicin and dihydrocapsaicin (conditions: column Betasil C_18_ (150 × 4.6 mm × 3 μm), mobile phase: H_2_O/CH_3_CN, 50:50 v/v, flow rate: 1.5 mL/min, UV detection at 222 nm).

**Figure 2:**
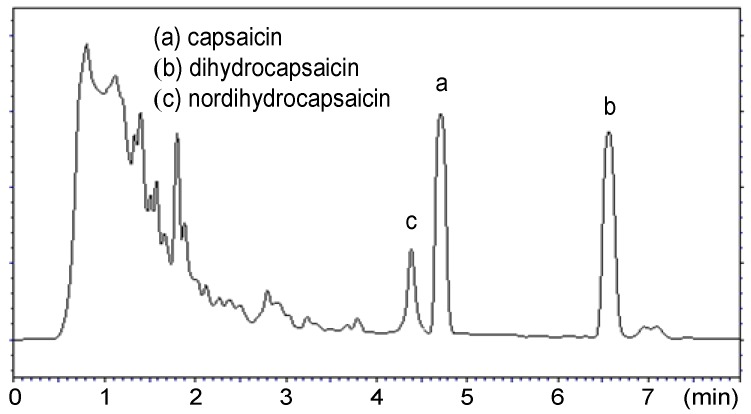
Chromatogram of the red chili extract (conditions: column Betasil C_18_ (150 × 4.6 mm × 3 μm), mobile phase: H_2_O/CH_3_CN, 50:50 v/v, flow rate: 1.5 mL/min, UV detection at 222 nm).

Extraction and quantitation steps were carried-out in duplicate for each sample. The standard solutions used for the calibration curve were regularly injected at intervals between sample injections to confirm the retention times. The chromatograms shown in Figure 1 and Figure 2 correspond to a standard and extracted solution, respectively; they reveal that capsaicin (a) and dihydrocapsaicin (b) are eluted at 4.69 and 6.51 min, respectively.

In all chromatograms obtained for the investigated peppers, the main identified peaks of interest corresponded to these two predominant capsaicinoids. On the other hand, it should be noticed that another peak (c) corresponding to nordihydrocapsaicin is also observed in all chromatograms.

**Figure 3:**
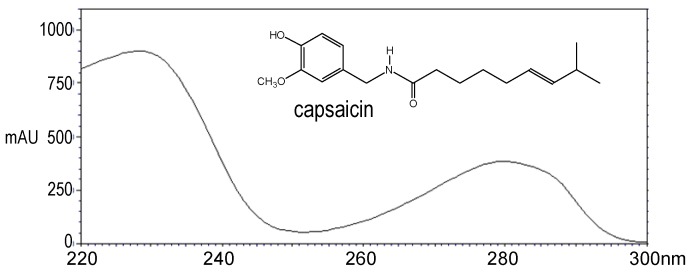
UV spectrum of capsaicin peak eluted at 4.69 mn. The maximum absorbance corresponds to λ_max_ = 228 nm.

**Figure 4:**
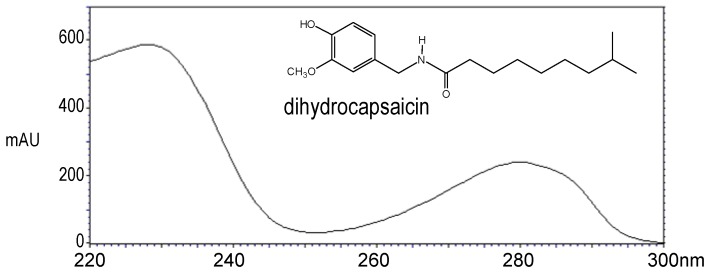
UV spectrum of dihydrocapsaicin peak eluted at t_R_ = 6.51 mn. The maximum absorbance corresponds to λ_max_ = 228 nm.

The ultraviolet absorption spectra corresponding to capsaicin and dihydrocapsaicin peaks were recorded from the photodiode array detector; they are shown in Figure 3 and Figure 4, respectively. For all quantitative measurements, the UV detection wavelength was set at 222 nm, because it corresponds to the maximum absorbance for both capsaicin and dihydrocapsaicin. On the other hand, the chromatograms showed a complete separation between capsaicin and dihydrocapsaicin and no interference with other capsaicinoid peaks.

The analytical method was validated by evaluating standard deviation, relative standard deviation, the limit of detection (LOD) and the limit of quantification (LOQ) for both capsaicinoids. The obtained results presented in Table 1 showed that the %RSD values were 1.01 and 0.57 for capsaicin and dihydrocapsaicin, respectively. The limits of detection (LODs) of the proposed method were 0.09 and 0.1 µg/g for capsaicin and dihydrocapsaicin, respectively; while the limits of quantification (LOQs) were 0.3 and 0.36 µg/g.

The data presented in Table 2 show the concentrations of capsaicin and dihydrocapsaicin, as well as the pungency expressed in Scoville heat units (SHU) in the analyzed pepper samples. The mean concentration of capsaicin in hot chili, red chili, green chili and green pepper was 4,249.0 ± 190.3, 309.3 ± 4.2, 138.5 ± 5.2, and 0.99 ± 0.9 µg/g respectively. On the other hand, the mean concentration of dihydrocapsaicin was 4,482.2 ± 35.6, 238.2 ± 2.6, 146.4 ± 4.2 in the first three samples, and it was not detected in green pepper. The highest pungency level evaluated in Scoville heat units (SHU) was observed with hot chili, while green pepper, red pepper and yellow pepper showed lower values. As shown in Table 3, the average daily capsaicin intake calculated for the population of Riyadh city was 7.584 mg/person/day.

Effective separation, identification and quantitation of the main characteristic capsaicinoids extracted from the different investigated pepper samples were possible by HPLC, as shown in Table 2. As mentioned in previous studies, the two most abundant capsaicinoids in peppers are capsaicin and dihydrocapsaicin [2].

The corresponding pepper contents obtained in µg/g were converted to Scoville heat units (Table 2) in order to classify them according to their various pungency levels. The results obtained showed that the concentrations of both capsaicin and dihydrocapsaicin in the peppers used for this study varied in the 0–4 mg/g range. The *Capsicum* green pepper, red pepper and yellow pepper had the lowest capsaicinoids content and the lowest pungency, as compared to hot chili and red chili. The contents of capsaicin and dihydrocapsaicin found in the present work for the different pepper varieties are in good agreement with those found by other authors who reported that a variation in capsaicin concentration is observed in the different peppers [30].

The results obtained in this study show that the contents of capsaicin (4,249.0 ± 190.3 µg/g) and dihydrocapsaicin (4,482.2 ± 35.6µg/g) in hot chili were higher than those recorded for cayenne pepper samples which had mean capsaicin and dihydrocapsaicin contents of 1,320 and 830 µg/g dry weight, respectively [25]. The use of the SHU parameter is the recommended method for pepper evaluation as it provides a better indicator of the pungency level, but it is considered less precise [31]. As shown in Table 2, our results showed that the pungency of the analyzed samples is in the following order: hot chili (highly pungent) > red chili (moderately pungent) > green chili (mildly pungent) > green pepper (non pungent) > red pepper and yellow pepper (non pungent).

The estimated pepper consumption by the population of Riyadh city (15.5 g/person/day) was higher than the values reported for India (2.5 g/person/day) and Thailand (5 g/person/day) [19] but lower than that calculated in Mexico (20 g/person/day) [19]. The daily capsaicin intake was 7.584 mg/person/day, which is higher than that for United States and Thailand, but lower than the value estimated for Mexico. Finally, there could be a risk for the population of Riyadh city from the consumption of the different peppers found in the local markets, as the daily capsaicin intake exceeds the maximum intake of capsaicin which was estimated to 2.64 mg/day [29].

## 3. Experimental

### 3.1. Samples, Chemicals and Solvents

**Figure 5:**
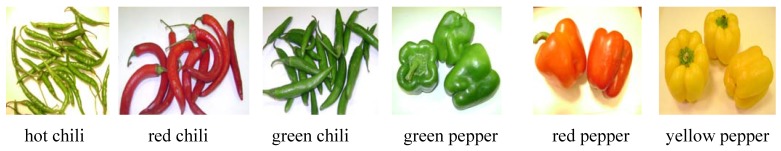
Pepper samples.

Food samples, including hot chili, red chili, green chili, green pepper, red pepper and yellow pepper, were purchased from local markets in Riyadh city (Figure 5). All these pepper types are varieties of *Capsicum annuum* L. and were imported fron India; the samples are preserved for any further investigation. Capsaicin and dihydrocapsaicin standards were purchased from Fluka Chemical Co. (Buchs, Switzerland). All solvents used as mobile phase were of HPLC grade and supplied by Aldrich (Steinheim, Germany).

### 3.2. Extraction of Capsaicinoids

All samples were first dried, then extracted using the method of Collins *et al.* [30] with slight modifications. For capsaicinoid extraction, each dried pepper sample (5 g) was placed in ethanol (5 mL) in a 120 mL glass bottle equipped with a Teflon lined lid. Bottles were capped and placed in a water bath at 80 °C for 4 hours, then swirled manually every hour. Samples were removed from the water bath and cooled to room temperature. The supernatant layer of each sample (5 mL) was filtered through 0.45 µm filter paper into a HPLC sample vial using a 5 mL disposable syringe (Millipore, Bedford, MA, USA). The vial was capped and stored at 5 °C in a refrigerator until analysis.

### 3.3. Liquid Chromatographic Analysis

The HPLC analyses were carried out on a Thermo HPLC system equipped with a Finnigan Surveyor Auto Sampler Plus, a Finnigan Surveyor LC Plus quaternary pump and a Surveyor photodiode array (PDA) detector. The chromatographic conditions were as follows:

- Betasil C_18_ column (particle size 3 μm, dimension 150 × 4.6 mm) from Thermo Electron (USA),
- column temperature: 60 °C, sample temperature: 20 °C, sample volume: 5 μL,
- UV detection wavelength at 222 nm,
- mobile phase: binary mixture water-acetonitrile (CH_3_CN) at a 50:50 ratio, flow rate: 1.5 mL/min.

**Figure 6:**
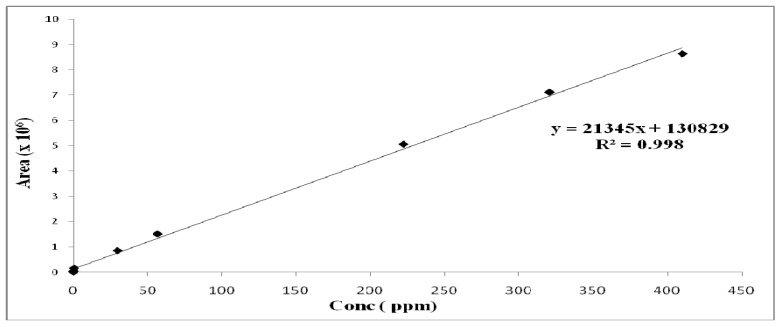
Calibration curve for capsaicin.

**Figure 7:**
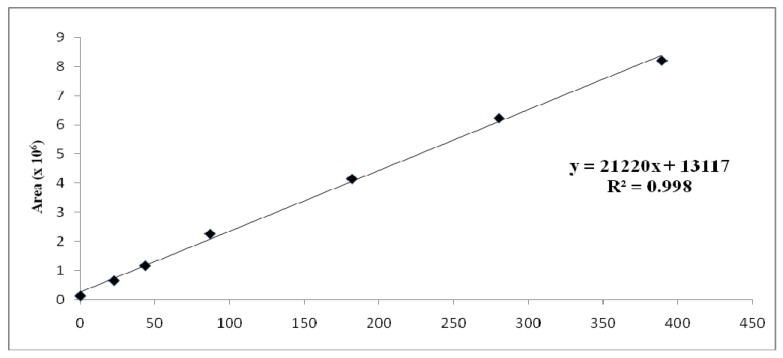
Calibration curve for dihydrocapsaicin.

The following standard solutions were prepared from a stock solution of capsaicin and dihydrocapsaicin using serial dilutions at 800, 600, 400, 200, 100, 50, 10, 1 and 0.5 µg/g. The standard solutions were run on the high performance liquid chromatograph and the obtained standard curve plots of peak area against concentration are shown on Figure 6 and Figure 7.

During HPLC sample analyses, a standard solution was injected every 10 samples in order to evaluate the retention time reproducibility and instrument calibration.

### 3.4. Capsaicinoids Quantitation

The major capsaicinoids in peppers, capsaicin and dihydrocapsaicin, were determined by comparison to external reference standards injected under the same conditions. Their identification was based on the retention times measured under identical HPLC conditions while their quantitative determination in the different peppers samples was carried out using the peak areas. The ratio between these capsaicinoids was calculated by dividing capsaicin and dihydrocapsaicin contents to the total capsaicinoids [22]. The capsaicinoid concentrations in samples are expressed as µg/g pepper.

### 3.5. Scoville Heat Unit Conversions

According to the commonly accepted Scoville organoleptic test, the spicy strength of the investigated samples was calculated by converting the capsaicin content expressed in grams of capsaicin per gram of pepper. This conversion to Scoville heat units was done by multiplying the capsaicin content in pepper dry weight by the coefficient corresponding to the heat value for pure capsaicin, which is 1.6 × 10^7^ [32].

### 3.6. Estimation of Dietary Capsaicin Intake

The average capsaicin intake per person per day was estimated from a 24 hrs food questionnaire which was distributed among two hundred and fifty families living in Riyadh city. The answers on the quantities of food they consumed regularly were collected and the mean values were calculated by multiplying the amount of each consumed pepper type by its mean capsaicin concentration; then the average daily intake was calculated as explained in the following example.

Supposing a daily consumption of two types of pepper of 2 and 3 grams, with a corresponding capsaicin concentration estimated to 1,000 µg/g and 500 µg/g, respectively; then the daily intake from each type will be obtained by multiplying the weight of each consumed pepper by its mean capsaicin concentration as follows:

daily intake from type 1 = 1,000 × 2 = 2,000 µg/day = 2 mg/day

daily intake from type 2 = 500 × 3 = 1,500 µg/day = 1.5 mg/day

The average daily intake of capsaicin (expressed in mg/person/day) will thus be:

= (2 + 1.5) = 3.5 mg/person/day

Assuming an average body weight of 70 kg, the daily capsaicin intake expressed in mg/kg body wt/day will be:

= 3.5/70 = 0.05 mg/kg body wt/day

## 4. Conclusions

Six *Capsicum* samples were investigated in order to determine their capsaicinoids composition. After extraction, the HPLC analyses allowed identification and determination of capsaicin and dihydrocapsaicin which were the main capsaicinoids in the different chili and pepper samples. Hot chili obtained from the local markets in Riyadh was the most pungent (67,984.60 SHU) among the peppers studied. The pungency of analyzed samples decreased as follows: hot chili (highly pungent), red chili (moderately pungent), green chili (mildly pungent), green pepper (non pungent), red pepper and yellow pepper (non pungent). The average pepper consumption by the population of Riyadh city and the corresponding daily capsaicin intake were 15.5 g/person/day and 7.584 mg/person/day, respectively.

## Figures and Tables

**Table 1:** Relative standard deviation, limits of detection (LOD) and quantitation (LOQ) for capsaicin and dihydrocapsaicin.

Parameter	Capsaicin	Dihydrocapsaicin
**Average peak area**	2221184.0	1617453.4
**SD**	22485.5	9286.3
**%RSD**	1.01	0.57
**LOD**	0.09	0.1
**LOQ**	0.3	0.36

**Table 2:** Concentrations of capsaicin, dihydrocapsaicin and Scoville heat units (SHU) in the analyzed pepper samples.

Pepper type	Capsaicin (µg/g)	Dihydrocapsaicin (µg/g)	Scoville heat units (SHU)	Levels of pungency
**Hot chili**	4249.0 ± 190.3	4482.2 ± 35.6	67984.60	highly pungent
**Red chili**	309.3 ± 4.2	238.2 ± 2.6	4949.08	moderately pungent
**Green chili**	138.5 ± 5.2	146.4 ± 4.2	2216.58	mildly pungent
**Green pepper**	1.0 ± 0.9	ND	15.83	non-pungent
**Red pepper**	ND	ND	0	non-pungent
**Yellow pepper**	ND	ND	0	non-pungent

ND: not detected.

**Table 3:** Average capsaicin daily intake in correlation with questionnaire.

Pepper type	Capsaicin (µg/g)	Daily consumption (g)	Capsaicin daily intake (mg/person/day)
Hot chili	4249.0	1.5	6.374
Red chili	309.3	3	0.928
Green chili	138.5	2	0.277
Green pepper	0.99	5	0.005
Red pepper	0	2	0
Yellow pepper	0	2	0
Average daily intake (mg/person/day)			**7.584**
Average daily intake (mg/kg body wt/day)			**0.108**
